# The acceptability and feasibility of a virtual mantram program for patients with posttraumatic stress disorder and substance use disorders: mixed method results

**DOI:** 10.1186/s12906-023-04312-1

**Published:** 2024-01-02

**Authors:** Sean Ferkul, Zena Agabani, Osamu Minami, Jill Bormann, Bernard Le Foll, Leah Lobo, Ahmed N. Hassan

**Affiliations:** 1 Department of Psychiatry, Campbell Family Mental Health Research Institute, Centre for Addiction and Mental Health, 100 Stokes Street, Third Floor, Toronto, ON M6J 1H4 Canada; 2 Department of Pharmacology and Toxicology, Faculty of Medicine, University of Toronto, Toronto, ON M5S 1A1 Canada; 3 Hahn School of Nursing and Health Sciences, San Diego, CA USA; 4 Beyster Institute of Nursing Research, San Diego, CA USA; 5 University of San Diego, San Diego, CA USA; 6 Department of Psychiatry, Faculty of Medicine, University of Toronto, Toronto, ON Canada; 7 Institute of Medical Sciences, University of Toronto, Toronto, ON M5S 1A1 Canada; 8 Waypoint Research Institute, Waypoint Centre for Mental Health Care, 500 Church Street, Penetanguishene, ON L9M 1G3 Canada; 9 Department of Psychiatry, King Abdulaziz University, Jeddah, Saudi Arabia

**Keywords:** Mantram, PTSD, Substance use disorder, Feasibility, Acceptability, Mindfulness

## Abstract

**Background:**

There is a need for expanded options for therapeutic interventions for patients with posttraumatic stress disorder (PTSD) and substance use disorder (SUD). The study aimed to examine evidence for the feasibility, safety, and acceptability of a virtual Mantram Repetition Program for adults with PTSD and SUD.

**Methods:**

This project utilized mixed-method design (explanatory sequential design) to collect quantitative and qualitative data to evaluate the program in terms of its feasibility and acceptability. The program took place over Webex, an encrypted virtual platform. The group ran over 8 weeks, was 90 min in length, and facilitated by two individuals per cohort. Each group had 4–5 participants given each group cycle.

The study used the Mantram Repetition Program which is a brief mindfulness based non-tramua focused group intervention.

**Results:**

Out of 43 participants enrolled, 5 people (11.6%) did not commence the program and 8 (18.6%) participants dropped out after commencing the program, resulting in 35 completers (81.4% retention rate). Treatment completion and retention were above 70%. Qualitative data explained several aspects of the program’s acceptability including delivery methods, informative material provided and gaining a practical mindful tool to manage symptoms.

**Conclusions:**

This study showed quantitative and qualitative evidence of the Mantram Repetition Program’s feasibility, acceptability and safety to be used with individuals with PTSD-SUD. Although further evaluation of virtual Mantram Program to control group in longitudinal trials is needed to identify how it compares with other interventions in the field.

**Clinical trial registration number:**

NCT05058963, (28/09/2021).

**Supplementary Information:**

The online version contains supplementary material available at 10.1186/s12906-023-04312-1.

## Background

Posttraumatic Stress Disorder (PTSD) is a debilitating disorder affecting individuals through multiple clusters of symptoms [1]. Epidemiological data suggests that PTSD and substance use disorder (SUD) are highly comorbid [2]. Among patients diagnosed with PTSD, the prevalence of comorbid substance uses ranges from 19 to 35%, and some substances (e.g., alcohol), ranges from 36 to 52% [3]. Several causal pathways and theoretical models have been studied to explain the bidirectional relationship between PTSD and SUD, subjecting individuals with PTSD to develop SUD and vice versa [4, 5]. While approximately half of individuals seeking SUD treatment meet the criteria for current PTSD, these individuals tend to have poorer treatment outcomes compared to those without such comorbidity [6]. Comorbid PTSD/SUD is associated with a more complex and costly clinical course when compared with either disorder alone [7].

Despite the major interruption of functioning which occurs among individuals with a PTSD and SUD comorbidity, there remain few effective treatments which can address these disorders, and these continue to evolve [8]. Trauma-focused therapies, such as prolonged exposure and cognitive processing therapy, and non-trauma focused therapies, such as Seeking Safety, has shown low rates of treatment completion in this population [9]. Furthermore, there are significant concerns that these therapies might trigger a relapse to substance use [10]. A limited availability of these therapies within the community may further perpetuate delays in receiving essential treatment. The substantial drop-out rate from these therapies also adds to the concern [11]. This is further complicated due to the nature of trauma being a social determinant of health. This makes accessing programming another challenge for individuals seeking support [12]. Therefore, it is integral to the field that there are expanded options for therapeutic interventions outside of traditional trauma therapies that is feasible and tolerable.

One of these options is the Mantram (singular: mantra) Repetition Program (MRP). The MRP is a mindfulness-based intervention with spiritual influences [13]. The program endeavors to enhance one’s concentration and attention through intentionally by silently repeating a self-selected mantram (spiritually based word or phrase), slowing-down thoughts and engaging in one-pointed attention. The MRP is a brief non-trauma focused spiritually based intervention. It has proven to be effective in reducing the recurrence of thoughts and emotions associated with PTSD [14]. With a focus on the ‘*mind–body-spiritual*’ technique, the MRP tools are perceived as relatively simple and practical [14]. The MRP has been found to be acceptable for variable populations, with good outcomes, including individuals with PTSD, chronic illnesses such as HIV and cancer, family caregivers, and health care professionals [15]. In addition, MRP interventions for soldiers experiencing PTSD saw meaningful reduction in insomnia and PTSD symptoms [16]. Though not directly identified, this reinforcement in structure and routine may play a role in encouraging additional treatment seeking. Therefore, potentially playing a positive role in identifying quality gaps that may exist and limit populations who have experienced PTSD in accessing structured therapeutic interventions in the future.

The features of the MRP hold potential as a treatment because of the intersection of skills integrated within the program. Specifically, offering clients an opportunity to practice skill building (e.g., one-pointed attention and mono-tasking), grounding, and mindfulness interventions that can support clients in their recovery at the initial phases of early treatment. These skills are necessary to help patients regulate their emotions specifically when triggered by trauma-reminders, craving for substances or both. Furthermore, MRP supports a novel treatment option for clients who have been unable to access trauma treatments due to their SUD or inability to tolerate trauma-focused therapy and offers a non-stigmatizing accessible treatment. Therefore, MRP allows for greater early engagement that can potentially impact continued support, such as CPT, in the future. MRP differs from any other kind of intervention because it is accessible and independent of the environment. Clients can engage in mantram anytime and anywhere. Also, many mindfulness interventions encourage clients to “turn inward, which can elevate distress in clients with PTSD who may view their bodily symptoms as triggers. Thus, clients can direct their attention towards the mantram rather than the body to ground themselves by encouraging focus on a word or phrase. Accessing care to support individuals with SUD and PTSD, in light of the COVID pandemic, has proven to be an extreme challenge. Furthermore, the application of MRP for individuals with SUD has not been examined until now. Therefore, the study aimed to examine qualitative evidence for the feasibility, safety, and acceptability of a virtual MRP for adults with PTSD and SUD.

## Methods

This project utilized mixed-method design (explanatory sequential design) to collect quantitative and qualitative data to evaluate the program in terms of its feasibility and acceptability. All participants received virtual treatment group and there was no comparison or control group.

### Virtual mantram program

Participants were placed into groups, each led by two facilitators and consisting of approximately 4–5 participants. There was a total of four facilitators leading the groups. Facilitators varied in their training background which included psychiatrist, psychologist, and social workers. Each group met weekly for approximately ninety-minute sessions over the 8-week program duration. All groups, and its assessments, were conducted via the Centre for Addiction and Mental Health (CAMH) WebEx platform. A summary of the program content (manualized) is presented in Supplementary Table 1. The content was adapted to address substance use and its cravings. Each participant received a hardcopy manual that highlighted the program’s skills and exercises.

### Study setting

This project’s research staff, recruitment process, and virtual group meeting were hosted at CAMH. CAMH is the largest mental health and addictions research center and teaching hospital in Canada, and one of the largest in the world.

### Study population

Treatment-seeking individuals were recruited through advertisement flyers and provider referrals within CAMH. The inclusion criteria were: i) participants 18-years old or older; ii) fluent in English; iii) diagnosis of PTSD confirmed with the Clinician-Administered PTSD Scale for DSM-5 (CAPS-5 past month version); iv) diagnosis of past-year substance use disorder other than caffeine by a healthcare practitioner confirmed by Structured Clinical Interview for DSM-5 (SCID-5); and v) agreed not to participate in other therapies during the program such as interventions including mindfulness, yoga, biofeedback, self-hypnosis, tai chi, 12 step, or one on one support) that might act as confounder. Exclusion criteria are: i) diagnosis of a severe or unstable medical illness that precludes safe participation in the study by a healthcare practitioner; ii) diagnosis of schizophrenia, schizoaffective disorder, or bipolar disorder; or current acute psychosis or mania by a healthcare practitioner; iii) participants with moderate or high risk of suicide upon screening by the Columbia Suicide Severity Rating Scale (C-SSRS) or v) have an inability to communicate in English fluently enough to complete the questionnaire.

### Measures

#### Demographic characteristics

Demographic characteristics were collected using the Addiction Severity Index (ASI) questionnaire at baseline assessment. It included age, gender, race, ethnicity, marital status, income, education, and occupational status.

### Retention

Retention was assessed by monitoring the number of sessions completed for each participant and number of participants who withdrew or dropped out after enrollment. Completers of the MRP were defined as attending at least six out of the total eight sessions, as defined by other researchers [16].

### Qualitative data collection

Following completion of the MRP, participants were invited for an optional focus group at approximately four-weeks post intervention. Focus groups were conducted to capture a more nuanced perspective on the unique features of MRP and ask questions that might explain acceptability or suggestions for improvement. The discussion questions presented during the focus group interviews included questions about the expectation and impression of the program, usefulness of learned skills and preference of delivery methods, presented in supplementary Table 2.

### Qualitative data analysis

We used descriptive qualitative analysis of the interview data from our focus groups to understand trial participation narratives among our sample. All data was coded independently, by two authors (A.H. and L.L.), in the ATLAS.ti computer assisted qualitative data analysis software platform to identify emergent patterns and key themes [17, 18]. Following Braun and Clarke’s guide on thematic analysis, the two authors organized codes into sub-themes and overarching themes [19]. A third author (S.F.) resolved any conflict in coding themes and confirmed overall important themes and agreement between the two coders. All quotations followed the institutional review board requirement of using pseudonyms to protect subject identities.

## Results

**Fig. 1:**
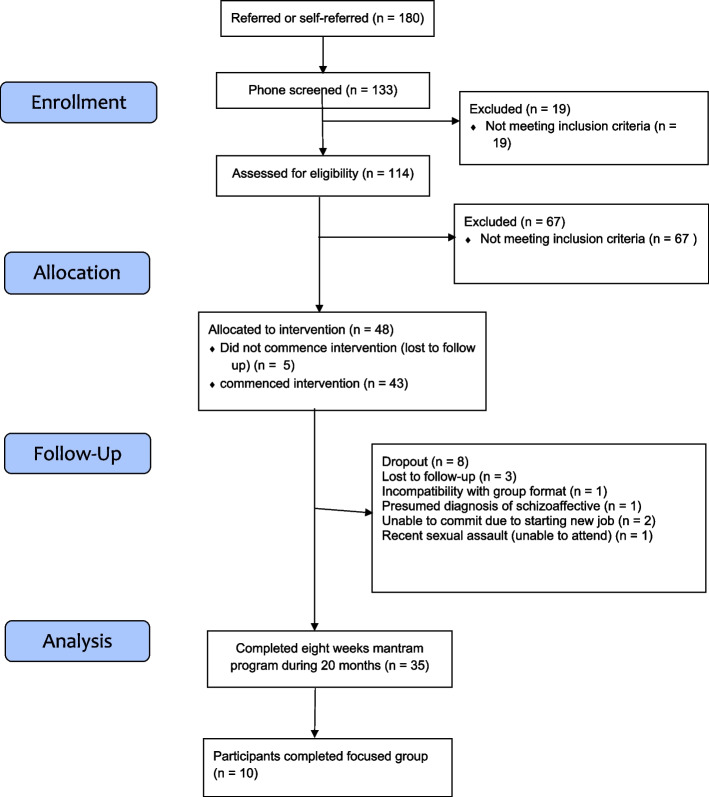
Recruitment flow diagram

During a 20 months period, eleven groups completed the program. Figure 1 demonstrates the number of participants included during enrollment, allocation, follow up and analysis. We received 180 referrals from clinicians and self-referral during the 20 months since the program initiation. A total of 48 participants were enrolled into the program from May 2021 to November 2022. Out of 48 participants enrolled, 5 people (10.4%) did not commence the program and 8 (16.7%) participants dropped out, resulting in 35 completers (81.4% retention rate). On average groups ranged from 3–5 members per group cycle. Out of the individuals commenced the program, a total of 30 participants (69.8%) attended 6 or more sessions, out of the 8 sessions of the program. The average number of sessions attended was 6 sessions (including dropouts). 28.6% of completers (*n* = 10) attended the optional focus group four weeks post program completion.

Table 1 describes the demographics and clinical features of the participants. Most were above 45 years old; 65.1% were females and 79.1% were employed full-time or part-time. Most of the participants were actively using substances prior to the start of the group (81.4%). The most common problematic substance was alcohol followed by cannabis then cocaine. Most participants were currently seeking psychiatric treatment from their physicians (74.4%). Out of those with active substance use, eight (18.6%) successfully achieved abstinence (reported no substance use) by the end of the program compared to their baseline. Eight individuals had been already abstinent from substances in the 30 days prior starting mantram program and two individuals, out of eight, relapsed to substance use while in the program.

**Table 1:** The demographic and clinical features of the recruited individuals

*N* = 43	N	% M SD
Education Completed		15.09 3.09
Age		41.79 10.94
18–29	6	14.0
30–44	16	37.2
45–64	21	48.8
65 +	0	0
Sex		
Male	15	34.9
Female	28	65.1
Race		
1- White (not Hisp)	30	69.8
2- Black (not Hisp)	4	9.3
3- American Indian	1	2.3
4- Alaskan Native	2	4.7
5- Asian/Pacific	2	4.7
6-Hispanic/Mexican	3	7.0
7-Hispanic-Puerto Rican	0	0
8-Hispanic-Cuban	0	0
9-Unknown	1	2.3
Religion		
1- Protestant	3	7.0
2-Catholic	9	20.9
3-Jewish	0	0
4-Islamic	0	0
5-Other	13	30.2
6-None	18	41.9
Employment status		
1-Full-time	20	46.5
2-Part-time (regular hours)	12	27.9
3-Part-time (irregular hours)	2	4.7
4-Student	0	0
5- Military Service	0	0
6- Retired/Disability	1	2.3
7- Unemployed	8	18.6
8- In controlled environment	0	0
Marital status		
1- Married	11	25.6
2-Remarried	0	0
3-Widowed	1	2.3
4-Separated	1	2.3
5-Divorced	9	20.9
6-Never married	21	48.8
Family history of depression	22	51.2
Family history of anxiety	18	41.9
Family history of substance use	17	39.5
Past 30-day substance use	35	81.4
Alcohol	27	61.4
Heroin	1	2.3
Methadone	1	2.3
Opiates	0	0
Barbiturates	0	0
Sedative	3	7.1
Cocaine	5	11.6
Cannabis	21	48.8
Hallucinogens	2	4.7
Inhalants	0	0
Amphetamine	1	2.3
Treated for psychiatric issue (past 30)	32	74.4
Controlled environment (past 30)	5	11.6

### Qualitative data results

**Fig. 2:**
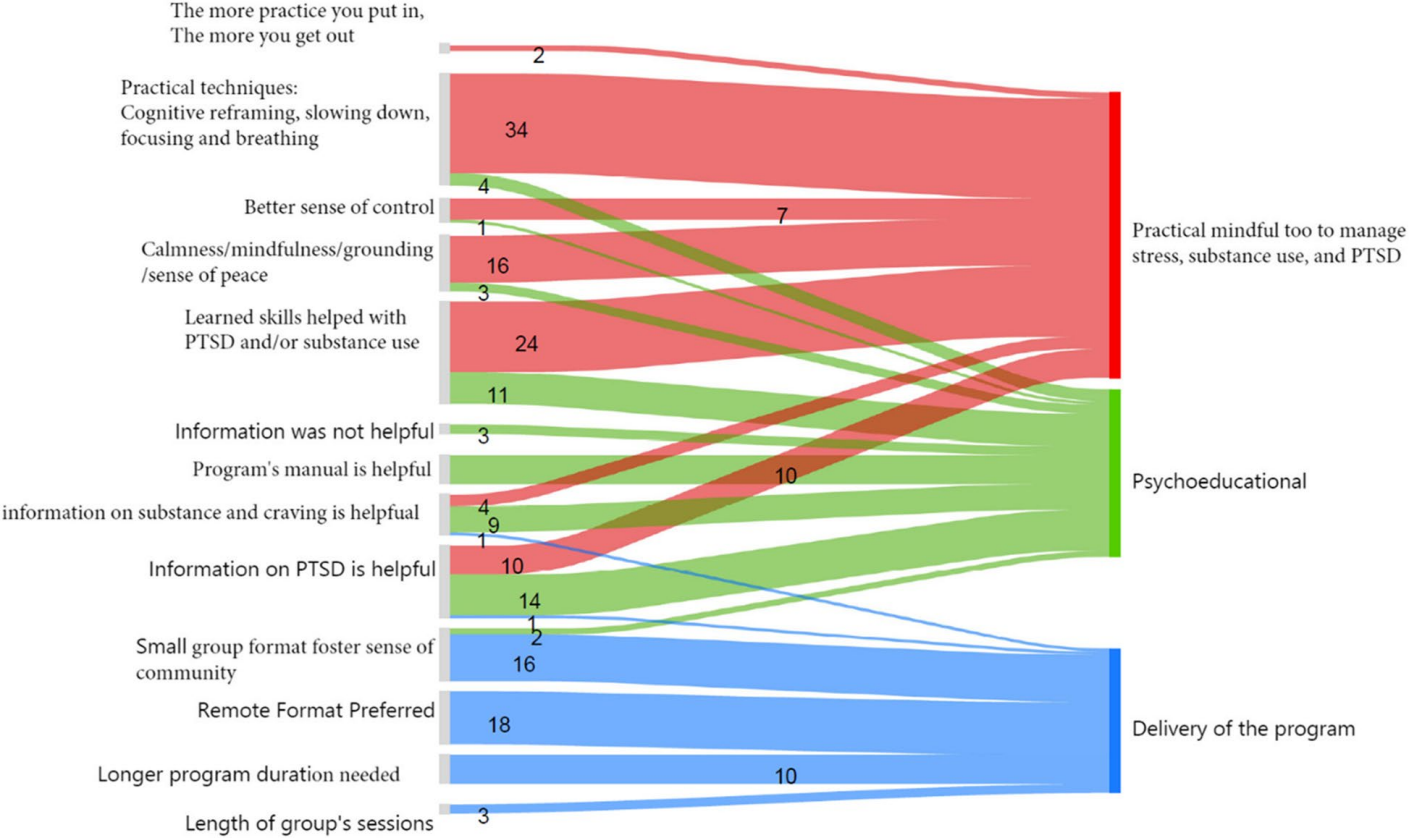
Diagram for the overarching themes and subthemes along with counts from participants completed mantram repetition program

There were several themes that highlighted the acceptability of the MRP. These themes included the virtual mode of delivery, new information, and tools gained to manage stress, PTSD and substances. Figure 2 visualizes the overarching themes and subthemes analyzed to highlight the feasibility and acceptability of MRP.

#### Delivery of the program

Participants reported that the virtual delivery of the program was both convenient and accessible. Generally, participants found the length of the group sessions to be ideal. On the whole, virtual delivery of the program helped ensure greater accessibility to facilitate inclusion of those who may be constrained by factors such as geographical remoteness, mobility issues, and financial hardship. One group member commented, “At least right now I prefer virtual because of COVID, not necessarily because of the other people in the group but to not have to take transportation every day and also, it’s nice to be home.” Another participant noted “It was nice to be virtual too because it is about PTSD and substance use discussion which can be triggering, so being at home in a safe environment was beneficial for myself”. This quote encapsulates some of the benefits that incorporating virtual care can have on feasibility and acceptability to participants entering the program to encourage trauma-informed responses of care that allow participants to engage in programming within a safe/home environment.

The small group format was described to foster a greater sense of community among participants. Participants in the focus group felt they strongly benefitted from hearing the experiences of others (including facilitators), noting “It was a lot of really useful information. The group itself was small enough that we got really good feedback and we were able to interact a lot with the facilitators and with each other. Yeah, I would say overall, I found it very helpful”. The small group format allowed for greater intimacy of the small attendance since it allowed for more depth to explore the Mantram tools and its applications. The small group size might have encouraged further discussion as one participant commented “I found the group very therapeutic and genuine, which enabled us to just have very open conversations”.

In contrast, there were differing opinions about the length of the program. Some participants indicated that a longer length of the program and greater follow-up resources may increase program feasibility (specifically to deal with substance use). One comment that spoke specifically to the length of the program reported “Yeah, I think maybe doing the course over 10 or 12 weeks and doing it in an hour each week instead, might be helpful. I also found an hour and a half to be long to be sitting at a computer and staring at the screen”. Whereas another participant highlighted “No, I don’t think 90 min was too long. For me, it was about the right time”.

### New information and resources

Participants highlighted the fact that they found the program’s manual and the educational materials provided at the beginning of the program to be informative in nature. Overall, participants found the SUD and craving information to be helpful; with some claiming to have benefitted especially from the information related to PTSD. A small minority of participants found the informational resources to be of little help in managing their PTSD symptoms and substance cravings. One individual commented that "the best thing [they could] say about what the program helps with, [was] the information that was shared.".

### Practical mindful tool to manage stress, substance use cravings and PTSD

Another theme that arose was that participants found that the development of a practical new skill (Mantram repetition) encouraged acceptability and engagement with the program. Most participants indicated mantram repetition was practical to be used anywhere, in contrast to most meditation techniques. One individual highlighted the “… skills around slowing down and taking the time to be reflective instead of reactive. Those were the major things for me”. This comment encapsulates one of the benefits noted by individuals around the benefits of developing a hard skill that supports and allows their development in slowing down reaction time and encouraging reflection around their responses to make decisions that better reflect their values and goals. Another individual noted that “it’s slowing me down so that I can process thoughts, so I can slow down and think about what I’m going to say, pause, so I’m not just jumping so much in that way”. The feedback here endorses a key theme and distinction between the MRP and other skills because it highlights that the Mantram is not a distraction tool, but rather one that allows the participant to engage with their environment in a safe and meaningful way.

In promoting mental grounding, Mantram repetition allowed participants to center their attention internally and be more aware of their own identity and surroundings. A common theme among participants was establishment of a sense of calm, something considered especially important when devolving into such states of agitation. In switching from what would otherwise be a high-stress scenario, the group proved exceedingly accessible. For example, one participant remarked, "It's [mantram repetition] sort of a good circuit breaker or a reset button or something like that, where I can just say okay, let's start fresh." Another participant said “It [repeating mantram] helped elevate my awareness and ground me in my body long enough to respond to situations in a way that honoured and dignified myself instead of reacting. So, it helped me live in my body and feel comfortable with the discomfort”.

In terms of managing substance cravings, the extra time given by the mantram to think objectively of one's circumstance prompted some participants to delay use and oftentimes avoid end up using at all. Through facilitating a ‘cognitive shift,' or 'cognitive reframing’; mantram repetition helped participants develop the internal skill of concentration, which they could gradually transfer externally to a variety of tasks. Another individual reported they delayed their cravings: “More so in … I would use the mantram for cravings and it would basically delay any use, and then sometimes I wasn’t using it at all, so compared to what I was using in the past, it definitely helped with that”. Another theme that was reported during the study was that mantram repetition helped participants to feel empowered in the management of their substance use goals. One participant reported “They [cravings] are way down, and I have got to say, yes, because they’re down, they [cravings] are way more manageable. So, I can bring in some other techniques too, to battle with that craving”.

A large majority of participants felt the benefit was contingent on the amount of effort put in. In other words, they reported that more practice of repeating their mantram, resulted in changes in their stress or clinical symptoms.

## Discussion

Individuals with comorbid PTSD/SUD are susceptible to high disability from these concurrent disorders. Previous studies has shown a significant role of mindfulness in mediating the relationship between PTSD symptoms and SUD severity [20]. However, systematic reviews and meta-analyses showed the mindfulness-based interventions for SUD has low effect size and low attrition and acceptability [21, 22]. Furthermore, systematic reviews on mindfulness-based interventions for PTSD showed medium to large effect size, but its effect on individuals with this comorbidity is unknown [23]. The studies that have looked at mindfulness interventions specific to concurrent PTSD-SUD showed potential benefits in terms of PTSD outcome but not substances’ craving and some were small in size and for specific sub-population (i.e., women only) [24–26]. There is a crucial need for the advancement of treatments focused on the unique needs of this comorbid group.

This is the first study to implement virtual MRP; a group-based, non-trauma focused program adapted for individuals with comorbid PTSD/SUD. This phase of the study focused on the feasibility and acceptability of the MRP within this population. This project showed feasibility and acceptability of MRP for this population with comorbidity due to its modified delivery format, psychoeducation materials, and modified tools that could be integrated into everyday routines, making it a valuable tool in dealing with both PTSD and SUD symptoms [27].

Feasibility benchmarks for this study included full recruitment in the allocated time-period, with 81.4% retention rate and 85.7% program completers (attended at least six out of eight sessions). Our attrition rate (18.6%) is consistent with the reported overall attrition rate of a systematic review of clinical trials that used mantram program (18%) [15]. A systematic review that looked into group-based trauma-focused therapies and non-trauma focused therapies for individuals with PTSD-SUD found a “low” treatment completion rate in both types of therapies (76.1% and 53.8%, respectively) [9]. Our study provided early evidence that virtual MRP is feasible, acceptable, and safe in individuals with PTSD-SUD with low risk of relapse.

Our focus group provided more details that explained these findings. The majority preferred virtual delivery of the program which gave them a greater sense of safety from COVID-19 as well as trauma-related safety. Literature has shown that participants who face challenges with PTSD are more susceptible to hypervigilance and perceptions of threats in their environment [28]. Therefore, virtual care may provide an option for engagement that rarely exists for in person therapeutic interventions for clients who experience PTSD. The small size of the group provided a sense of cohesiveness and provided more collaboration between facilitators and group members. This resulted in reduced barriers to accessing and engaging in the program. Our results also showed that integrating some psychoeducation material about PTSD and/or SUD was beneficial for individuals approaching treatment. This is consistent with a randomized trial that found effectiveness on PTSD outcomes from a single psychoeducation session [29]. The integration of psychoeducation in this program, which is also present in other trauma focused and non-trauma focused therapies, has enhanced acceptability of the program. Participants appreciated the availability of hardcopy handbook that reminded them to practice their skills. Modifications and adaptations in terms of delivery methods could be made to existing therapies to support flexibility when engaging patients with comorbid PTSD and substance use challenges.

A lot of participants similarly found this meditation technique a helpful tool for their mindfulness-based stress management toolkit, useful in calming emotions while maintaining focus, mindfulness, and compassion in a wide variety of different scenarios [27]. Within the MRP there are several components that seek to target trauma and substance use symptoms. Specifically, the skills of slowing reaction time, monotasking, and directed attention are integrated within the program to target trauma and substance use management symptoms. For instance, it has been highlighted that patients with SUD have shown to have challenges with reward functioning [30] and quicker response times to stimulants such as alcohol [31]. Thus, one explanation of the acceptability of the program was the impact that it had directly on their ability to “slow down” their reaction time and evaluate the potential consequences and benefits of behaviors more thoroughly. This program was accepted well by patients because it provided a practical skill that allowed and supported an accessible tool to ground and engage in the world in a meaningful way. Providing expanded options such as the virtual MRP holds a systemic trauma informed response by creating another option for patients in accessing care when seeking support for their PTSD/SUD. This may have provided alternatives for patients that may feel limited in their choices for trauma-based treatments.

The main limitation of this study is the absence of a control group. Future studies should consider comparing virtual MRP to other available alternative therapies (PTSD-focused, SUD-focused or both). A meta-analysis showed that neither trauma-focused groups nor non-trauma focused group is significantly different in treatment completion compared to their comparator [32]. Therefore, there is a need to further evaluate controlled interventions in clinical trials to assess their capacity for treatment retention which is crucial for good outcome in this vulnerable population. The absence of efficacy measures on PTSD and SUD makes it difficult to confirm the association between acceptability and effectiveness.

## Conclusions

In summary, this study is the first to implement an adapted virtual MRP as a complementary treatment for individuals with PTSD and SUD. This study showed quantitative and qualitative evidence of its feasibility, acceptability and safety to be used in this treatment population. Treatment completion and retention were above 70%. Therefore, virtual MRP is a promising non-trauma focused treatment option that is feasible, acceptable and well tolerated for patients with PTSD-SUD. Further evaluation of MRP to control group in longitudinal trials is needed.

### Supplementary Information

**Additional file 1.**

## Data Availability

The dataset generated and/or analyzed during the current study are available from the corresponding author on reasonable request.
