# Supplementary material for: The acceptability and feasibility of a virtual mantram program for patients with posttraumatic stress disorder and substance use disorders: mixed method results
Source: BMC Complement Med Ther. 2024 Jan 2;24:9. doi: 10.1186/s12906-023-04312-1 (PMC10759462; doi:10.1186/s12906-023-04312-1)
Supplement: Supplementary file 1 — Additional file 1. [file 12906_2023_4312_MOESM1_ESM.docx]

| Week | content |
| --- | --- |
| 1 | Define and describe the characteristics of a “mantram” as taught by Eknath Easwaran and how it relates to posttraumatic stress (PTSD) |
| 2 | Identify ways to choose and use mantram repetition for training attention and targeting craving.  Introduction to substance craving and identify triggers for use. |
| 3 | Describe the relationship between the PTSD/stress response, mantram repetition and the “relaxation response” |
| 4 | Describe at least two benefits of slowing down versus automatic pilot for stress reduction |
| 5 | Describe at least two benefits of one‐pointed attention versus multitasking for stress reduction |
| 6 | Demonstrate how one‐pointed attention and slowing down complement each other for making healthy choices |
| 7 | List at least five strategies for making mantram repetition a part of your life |
| 8 | Discuss applications of mantram repetition, one‐pointed attention and slowing down for overall health and substance use |

Supplementary Table 1: Summary of mantram repetition program’s content.

| Question 1: What are your impressions of the group you completed? |
| --- |
| Question 2: Did it meet your expectations? Why or why not? |
| Question 3: What are some skills you learned throughout this program? |
| Question 4: Did you find the skills you learned effective in coping with your PTSD symptoms? If yes, in what way? If not, why not? |
| Question 5: Did you find this group useful in helping you in your struggle with cravings and  substance use?  a) In what ways was it useful?  b) In what ways was not useful? |
| Question 6: Is there anything you wish existed to help you that was not addressed in the program? |
| Question 7: Do you prefer the virtual delivery of this program over in-person meetings? Why or why not? |

Supplementary Table 2: Questions of the focus group at week 12 post start of Mantram Repetition Program
